# Transcutaneous bilirubin reliability during and after phototherapy depending on skin color

**DOI:** 10.1007/s00431-024-05516-4

**Published:** 2024-04-06

**Authors:** Júlia Candel-Pau, Silvia Maya-Enero, Jordi Garcia-Garcia, Xavier Duran-Jordà, María Ángeles López-Vílchez

**Affiliations:** 1 Department of Neonatology, Service of Pediatrics, Hospital del Mar, Universitat Pompeu Fabra, Parc de Salut Mar, Passeig Marítim 25-29, 08003 Barcelona, Spain; 2 AMIB (Methodological and Biostatistical Consultancy), IMIM (Hospital del Mar Institute for Medical Research), Doctor Aiguader 88, 08003 Barcelona, Spain

**Keywords:** Neonatal jaundice, Serum bilirubin, Hyperbilirubinemia, Transcutaneous bilirubin, Skin color, Phototherapy

## Abstract

Measurement of transcutaneous bilirubin (TcB) is a non-invasive, widely used technique to estimate serum bilirubin (SB). However, its reliability in multiethnic populations during and after phototherapy is still controversial even in covered skin. The aim of this study was to determine the reliability of TcB in covered (cTcB) and exposed (eTcB) skin during and after phototherapy in a multiethnic population of term and preterm neonates according to Neomar’s neonatal skin color scale. Prospective, observational study comparing SB and TcB. We determined SB when clinically indicated and, at the same time, measured cTcB under a photo-opaque patch and eTcB next to it with a jaundice meter (Dräger JM-105TM). All dyads TcB-SB were compared, both globally and according to skin color. We obtained data from 200 newborns (color1: 44, color2: 111, color3: 41, color4: 4) and compared 296 dyads TcB/SB. Correlation between cTcB and SB is strong during (0.74–0.83) and after (0.79–0.88) phototherapy, both globally and by color group. The SB-cTcB bias depends on gestational age during phototherapy and on skin color following phototherapy. The correlation between eTcB and SB during phototherapy is not strong (0.54), but becomes so 12 h after discontinuing phototherapy (0.78).

*  Conclusions*: Our study supports the reliability of cTcB to assess SB during and after phototherapy, with differences among skin tones after the treatment. The use of cTcB and Neomar’s scale during and mainly after phototherapy may help reduce the number of blood samples required.

**Table:** 

**What is Known:** *• Controversies exist on the reliability of jaundice meters during and after phototherapy in covered skin. Only a few studies have analyzed their accuracy in multiethnic populations, but none has used a validated neonatal skin color scale.*	
**What is New:** *• We verified correlation between serum and transcutaneous bilirubin in covered skin in a multiethnic population depending on skin color based on our own validated neonatal skin color scale during and after phototherapy.*

**Supplementary Information:**

The online version contains supplementary material available at 10.1007/s00431-024-05516-4.

## Introduction

Neonatal jaundice affects 60–85% of term infants, with pathological jaundice being the most common cause of readmission [1, 2]. It is potentially dangerous due to acute and chronic bilirubin encephalopathy [3]. The gold standard to measure serum bilirubin (SB) levels is by means of a blood sample and, for patients under phototherapy, it means frequent blood draws which causes pain and increases the risk of anemia and infections [3]. For these reasons, non-invasive methods like transcutaneous bilirubin (TcB) measurement have been proposed for patients undergoing phototherapy or after discontinuing it. Several authors have found a good TcB/SB correlation (0.78–0.96) before phototherapy, but controversies remain as to its reliability during and after phototherapy [4–14]. Their reliability seems to be also influenced by skin color, gestational age, TcB device, measurement site and TSB level [4–7, 9, 11–15]. Newborns in our reference area belong to multiethnic populations with different skin tones. Although TcB/SB correlation seems to be good regardless of skin color, TcB tends to overestimate SB to a higher degree in dark-skinned neonates [3, 4, 11, 16]. Jaundice meters may be less reliable in neonates receiving phototherapy or after interrupting it, but studies have found conflicting results [10, 13, 17–23]. Most studies found the correlation to be better when TcB was measured in covered skin, however most of them were not multiethnic [18, 20, 22, 24–28].

We decided to conduct this study to test the correlation between SB and TcB, both in covered (cTcB) and in exposed skin (eTcB), during and after phototherapy in a multicultural population including preterm babies, with the aim of reducing physical discomfort to the newborn, as well as economic costs. A previous study we conducted showed that the TcB/SB correlation before phototherapy depended on skin color as determined by Neomar’s neonatal skin color scale but not on gestational age [29]. Our hypotheses were that cTcB correlated well with SB during phototherapy and immediately afterwards, however eTcB did not until 24 h after discontinuation of phototherapy, with a significantly different bias depending on skin color.

## Methods

This was a prospective observational study conducted at the neonatal unit of a tertiary care center with approximately 1400 deliveries/year which compared the levels of bilirubin using two techniques: TcB and SB. All the neonates admitted to our hospital with indication of phototherapy during the study period (October 2016 to March 2019) were offered to participate. The indication of phototherapy was based on SB according to our hospital guidelines [30, 31]. Patients were enrolled if their parents agreed to and signed a written informed consent. Exclusion criteria were the presence of extensive skin lesions in the sternal zone. None of the participants had been under phototherapy when first recruited. We did not exclude patients who had ABO/Rh incompatibility. We included preterm neonates because they are more prone to requiring phototherapy and thus need more bilirubin measurements.

Neonates in our hospital are routinely assigned to a color group at 24 h of life according to Neomar’s skin color scale (4 categories: light/color1, medium-clear/color2, medium-dark/color3 and dark/color4) (see Appendix 1) [29]. We also routinely measure TcB in the mid-sternal area as recommended by the manufacturer (Dräger Jaundice Meter JM-105™, Minolta, Dräger Medical GmbH, Lübeck, Germany) for the screening of neonatal jaundice. Our laboratory determines SB with a diazo method (COBAS INTEGRA® 400 plus analyzer, Roche Diagnostics).

Prior to the initiation of phototherapy, we covered a mid-sternum area with a 26-mm-photo-opaque patch (NeoSmile™ temperature probe cover, NEOTECH PRODUCTS, USA) that we kept on during phototherapy and after its discontinuation until a normal rebound SB measuring was obtained.

Phototherapy was provided continuously by standard overhead phototherapy units (Dräger Photo Therapy 4000 with 4 lamps, Dräger Medical GmbH, Lübeck, Germany) placed 35–40 cm above the neonate, which delivered irradiation in a central area of 1.6 ± 0.3 microWatts/cm2. The lamps (Draeger Fluorescent light “blue” 2 M 21 010) deliver blue light (spectral wavelength: 400-550 nm, peak: 450 nm). During phototherapy, the infants’ eyes were routinely covered by opaque eye pads that were regularly checked by nursing personnel at 3 h intervals during feeds. Phototherapy was only discontinued during TcB or SB measurements. The cTcB measurement was performed under the photo-opaque patch on the covered mid-sternal area, and the eTcB measurement on the adjacent exposed area. Paired cTcB/SB and eTcB/SB dyads were taken within 20 min of each other.

We collected data on gestational age, gender, prematurity, mother’s country of origin (surrogate for the participant’s ethnicity), birth weight, feeding choice, paired eTcB/SB and cTcB/SB (see Data collection sheet, Appendix 2).

Statistical analyses: We described quantitative variables (gestational age, birth weight, cTcB, eTcB and SB), which were normally distributed, using the mean and standard deviation (SD), and categorical variables (gender, prematurity, breastfeeding, mother’s origin/ethnicity) with frequencies and percentages. We compared the four-color groups in terms of gestational age, prematurity rate, birth weight, sex, mother's origin and rate of breastfeeding. Qualitative variables were compared with the Pearson’s chi-squared or Fisher’s exact test as appropriate and quantitative variables with ANOVA. During and after phototherapy, paired cTcB/SB and eTcB/SB measurements were compared, both globally and according to skin color and prematurity. We assessed the association between TcB and SB with Pearson’s correlation coefficient. Bland Altman plots were used to evaluate the concordance between techniques (cTcB/eTcB and SB). Mean difference (bias) and its limits of agreement (LOA) were reported for the whole sample and for each color group. Color3 and color4 participants were assembled in a unique group (color3or4) due to the small color4 sample and the lack of statistically significant differences between color3 and color4 SB-cTcB biases in our previous study [11]. We also performed a multiple linear regression to check the relation between SB-cTcB bias and cTcB measurements as well as gestational age and skin color group. The choice of these potential confounders was based on clinical criteria. These analyses were performed separately at each observation stage (during phototherapy and after phototherapy). Results from these analyses were expressed as β coefficient, its 95%CI and p-value. Significance was set at the p < 0.05 level. To perform statistical analyses we used STATA version 15.1 (StataCorp, College Station, TX, USA).

Our hospital Ethics Committee (“Comité de Ética de la investigación con medicamentos Parc de Salut Mar”) accepted and approved this study (ref. 2015/6519/I), which was conducted following the principles of the Helsinki Declaration.

## Results

We recruited 200 neonates who required phototherapy during the study period (color1: 44 patients, color2: 111 patients, color3: 41 patients, color4: 4 patients). Table 1 describes characteristics of our population. There were no differences among the color groups in terms of gender and rate of breastfeeding at discharge. However, there were statistically significant differences in terms of gestational age, prematurity, birth weight and mother’s origin. Jaundice was idiopathic in 93 neonates (46.5%), prematurity-related in 81 (40.5%) and due to ABO-isoimmunization in 26 (13%).

**Table 1:** Characteristics of our study population

	Global (n = 200)	Color 1 (n = 44)	Color 2 (n = 111)	Color 3 (n = 41)	Color 4 (n = 4)	p
**Gestational age** (weeks) (mean, SD)	37.25 (2.3) [30-41.7]	35.79 (2.6) [30-41.4]	37.36 (2.1) [33.1-41.7]	38.38 (1.8) [33.6-41.1]	38.64 (2.8) [34.6-40.6]	**<0.001**^a^
**Gender, female,** n (%)	92 (46)	25 (56.8)	48 (43.2)	18 (43.9)	1 (25)	0.362^b^
**Birth weight** (g) (mean, SD)	2757.04 (625.54)	2477.07 (703.34)	2782.39 (554.85)	2941.22 (621.39)	3245.50 (688.47)	**0.005**^a^
**Prematurity,** n (%)	81 (40.5)	29 (65.9)	44 (39.6)	7 (17.1)	1 (25)	**<0.001**^b^
**Breastfed, ** n (%)	195 (97.5)	41 (93.2)	109 (98.2)	41 (100)	4 (100)	0.192^b^
**Mother’s origin,** n (%) -Subsaharian -Latin American -Caribbean -East Asian -European -Magrebin -Southeast Asian	1 (0.5) 26 (13.0) 5 (2.5) 12 (6.0) 67 (33.5) 19 (9.5) 70 (35.0)	0 (0.0) 4 (9.1) 0 (0.0) 1 (2.3) 32 (72.7) 5 (11.4) 2 (4.5)	0 (0.0) 18 (16.2) 2 (1.8) 8 (7.2) 34 (30.6) 13 (11.7) 36 (32.4)	0 (0.0) 3 (7.3) 2 (4.9) 3 (7.3) 1 (2.4) 1 (2.4) 31 (75.6)	1 (25.0) 1 (25.0) 1 (25.0) 0 (0.0) 0 (0.0) 0 (0.0) 1 (25.0)	**<0.001**^b^

*TcB* transcutaneous bilirubin, *SB* serum bilirubin, *SD* standard deviation

^a^ANOVA

^b^Chi-squared test

We analyzed 296 paired SB/cTcB and 291 paired SB/eTcB measurements. Just before initiating phototherapy, the mean SB was 15.3 mg/dL (7.6–27.8 mg/dL), and the mean TcB was 15.86 mg/dL (8.8–20 mg/dL).

The mean age at initiation of the first phototherapy course was 68.04 (SD 37.18) hours and the mean duration of the phototherapy courses was 21.6 (SD 7.9) hours. A single phototherapy course was sufficient for 172 children, whereas 28 required two courses, and only 2 required three.

**Fig. 1:**
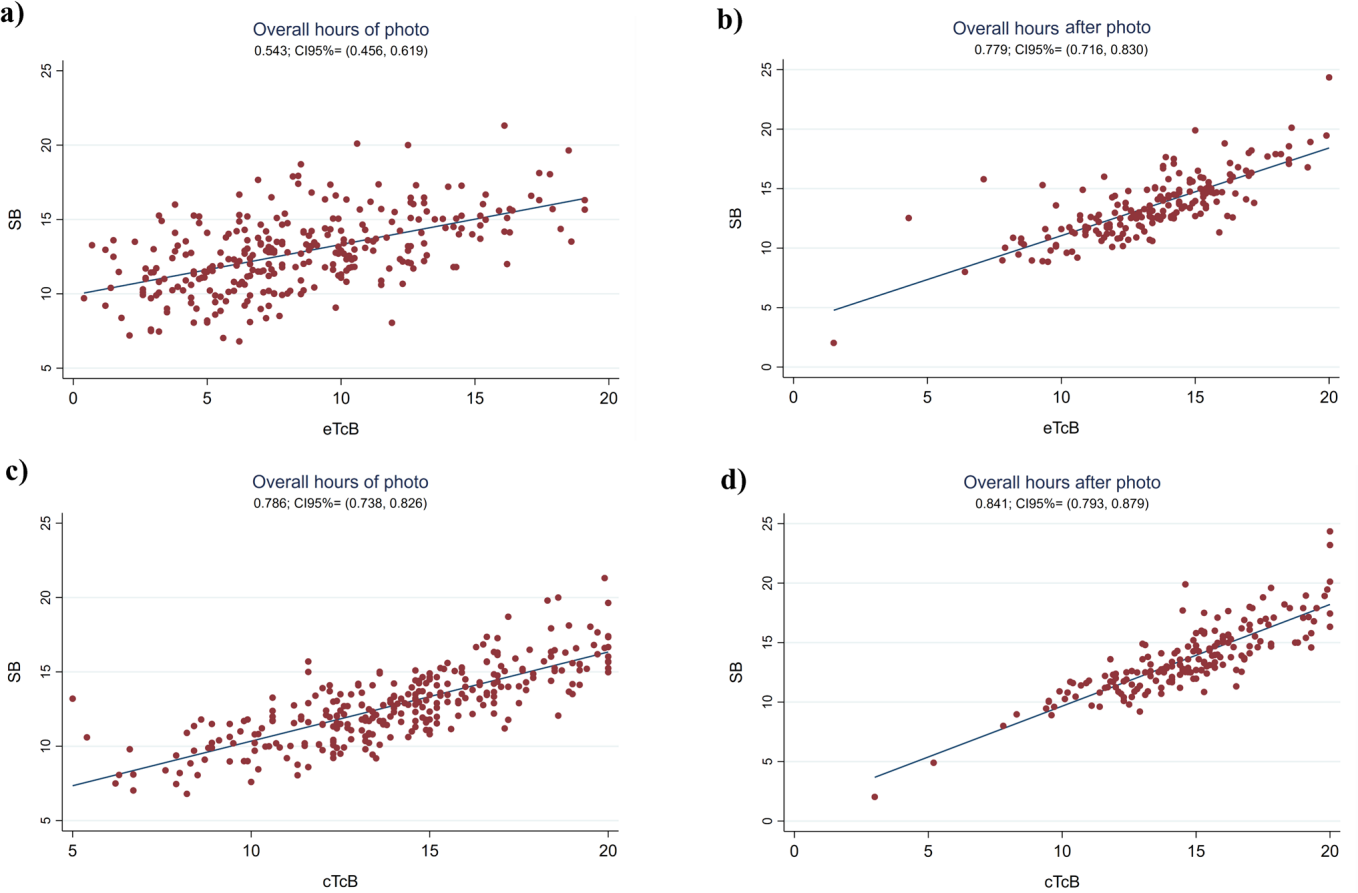
Pearson correlation coefficients. **a**. Between eTcB and SB globally during phototherapy (n = 291). **b**. Between eTcB and SB globally after discontinuing phototherapy (n = 186). **c**. Between cTcB and SB during phototherapy globally (n = 296). **d**. Between cTcB and SB after discontinuing phototherapy globally (n = 184)

**Fig. 2:**
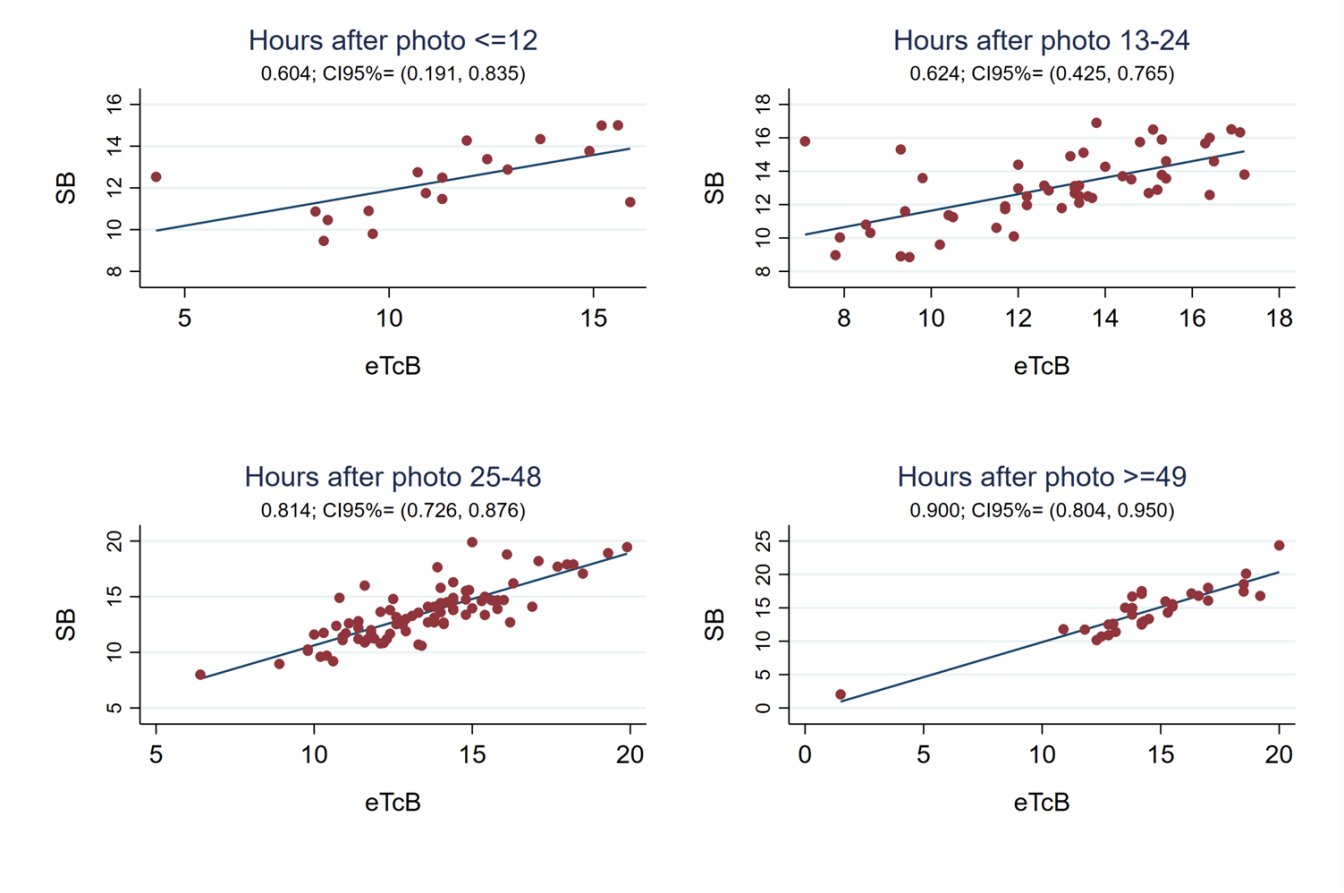
Pearson correlation coefficient between eTcB and SB after phototherapy depending on the hours after discontinuing phototherapy (first 12 h –n = 18-, 13 to 24 h –n = 53-, 25 to 48 h –n = 83-, and after 48 h –n = 32-)

The Pearson correlation coefficient between eTcB and SB was weak during phototherapy (r0.54, 95%CI 0.46;0.62) (see Fig. 1), with the SB-eTcB bias Bland Altman estimation of 4.31 mg/dL (95%LOA -2.46;11.07). However, the correlation improved globally following discontinuation of phototherapy to 0.78 (95%CI 0.72;0.83), still being weak the first 24 h (r 0.60–6.62) but significantly improving afterwards (r0.81–0.90) (see Figs. 1 and 2). The Bland Altman bias estimation and its LOA between eTcB and SB globally measured after phototherapy resulted in 0.18 mg/dL (95%LOA -3.42;3.78).

**Fig. 3:**
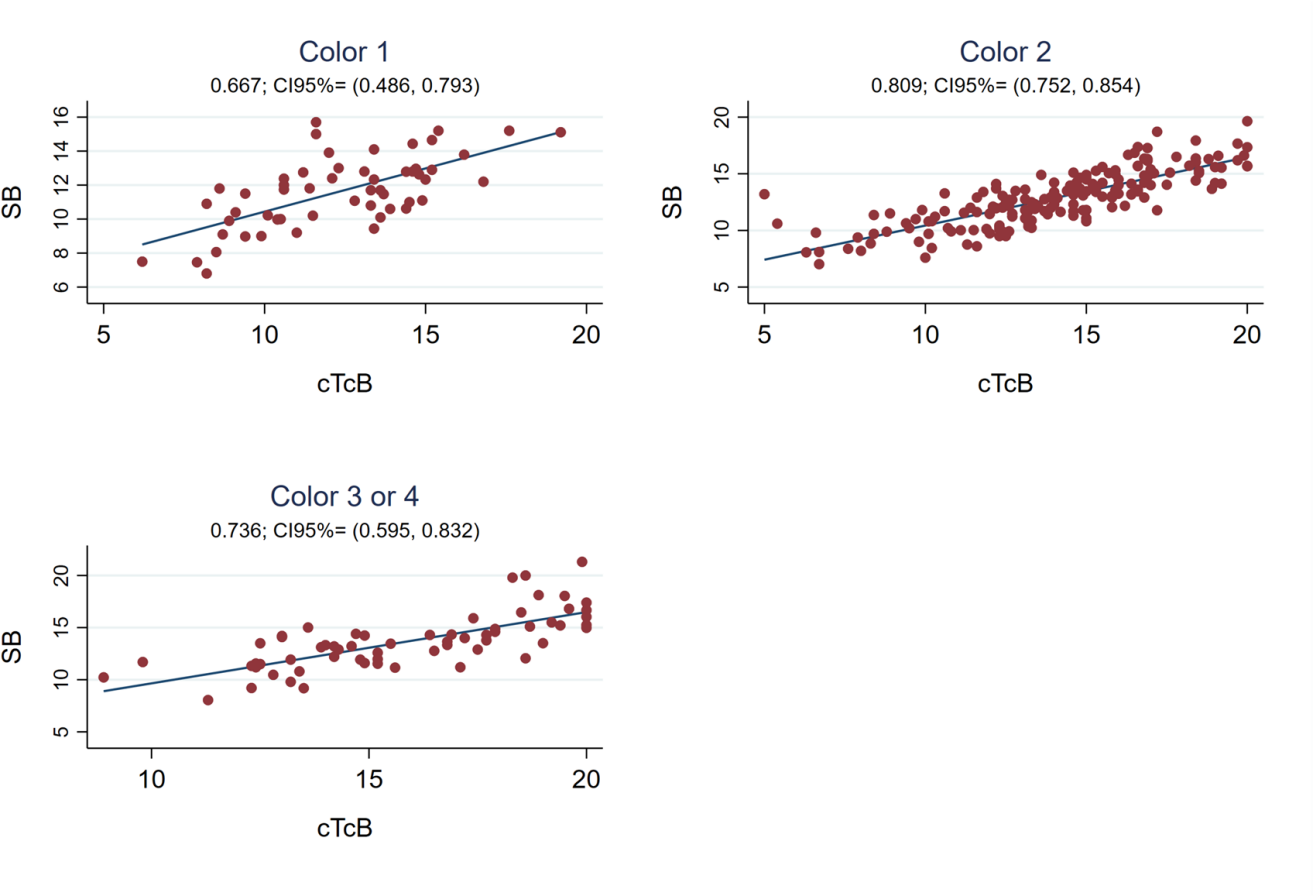
Pearson’s correlation coefficients between cTcB and SB during phototherapy depending on skin color (n = 54 for color 1, n = 180 for color 2, n = 62 for color 3or4)

**Fig. 4:**
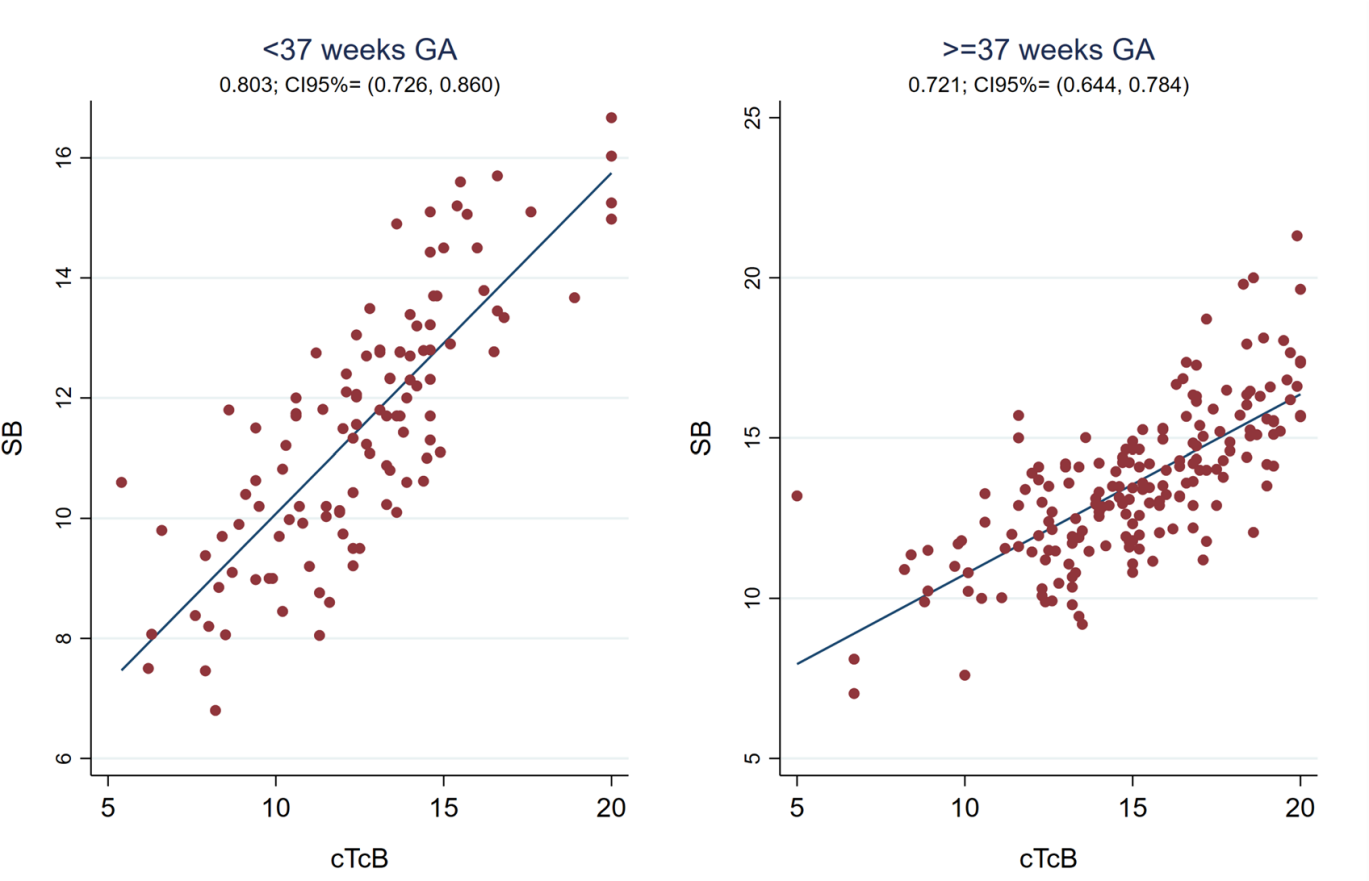
Pearson correlation coefficient between cTcB and SB depending on gestational age during phototherapy (n = 112 for < 37 GA, n = 184 for ≥ 37 GA). *GA: gestational age*

The global Pearson correlation coefficient between cTcB and SB was strong during phototherapy (r0.79, 95%CI 0.74;0.83) (see Fig. 1), with the SB-cTcB bias Bland Altman estimation of -1.30 mg/dL (95%LOA -5.24;2.64). Figure 3 shows the Pearson’s correlation coefficients between cTcB and SB during phototherapy depending on skin color: better for color2 (r 0.81) than for color3or4 (r 0.74) or color1 (r 0.67). Bland Altman bias estimation and its LOA between cTcB and SB depending on skin color during phototherapy resulted in: -0.70 mg/dL (95%LOA -4.77;3.36) for color1, -1.17 mg/dL (95%LOA -4.90;2.56) for color2, -2.20 mg/dL (95% LOA -6.13;1.72) for color3or4. Figure 4 shows the Pearson’s correlation coefficients between cTcB and SB during phototherapy depending on gestational age: stronger for preterm (r0.80) than for term infants (r0.72). Bland Altman bias estimation and its LOA between cTcB and SB depending on gestational age during phototherapy resulted in: -1.03 mg/dL (95%LOA -4.58;2.51) for preterm infants, and -1.46 mg/dL (95%LOA -5.60;2.68) for term infants.

**Fig. 5:**
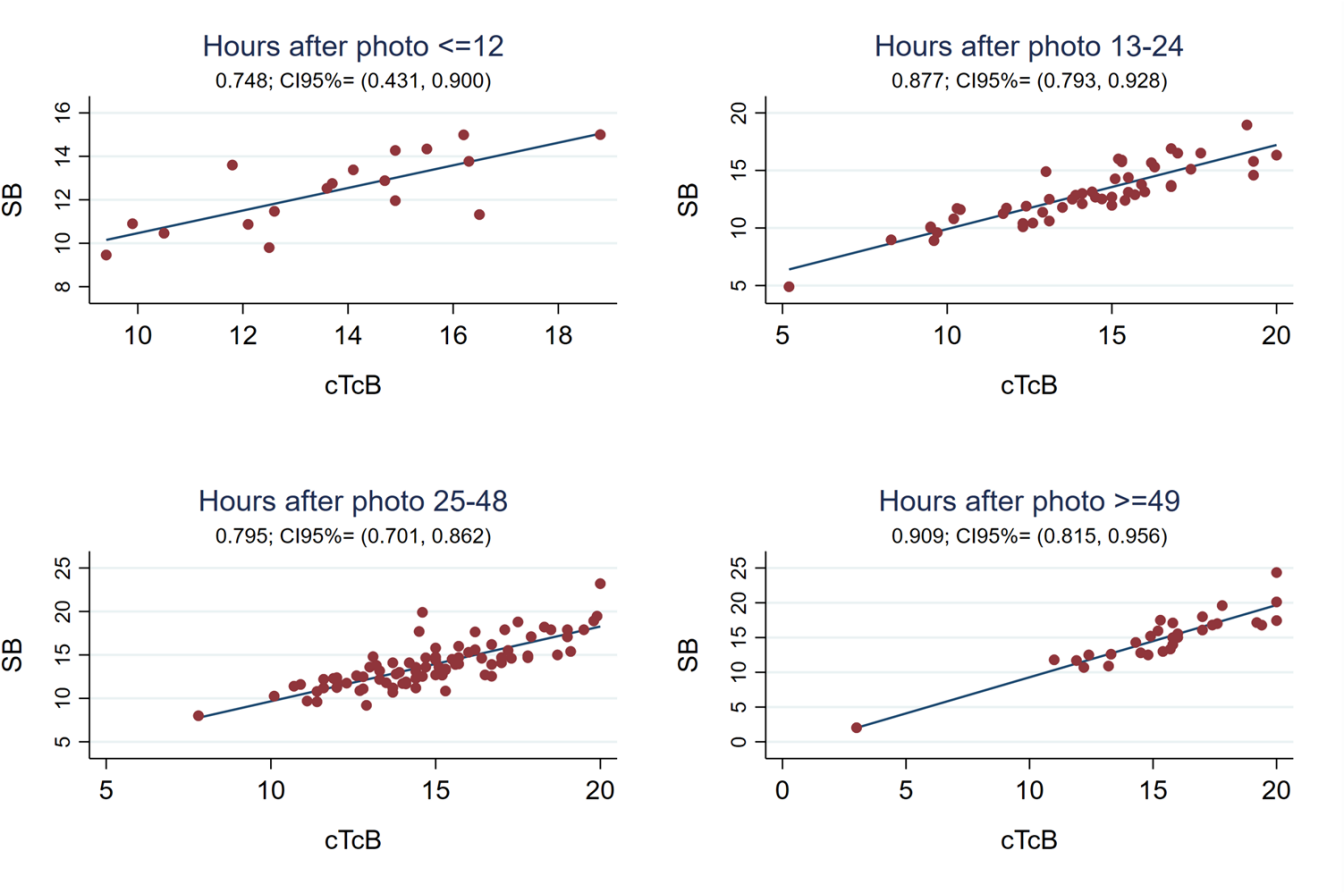
Pearson correlation coefficient between cTcB and SB after phototherapy depending on the hours after discontinuing phototherapy (first 12 h –n = 18-, 13 to 24 h –n = 51-, 25 to 48 h –n = 85-, and after 48 h –n = 30-)

**Fig. 6:**
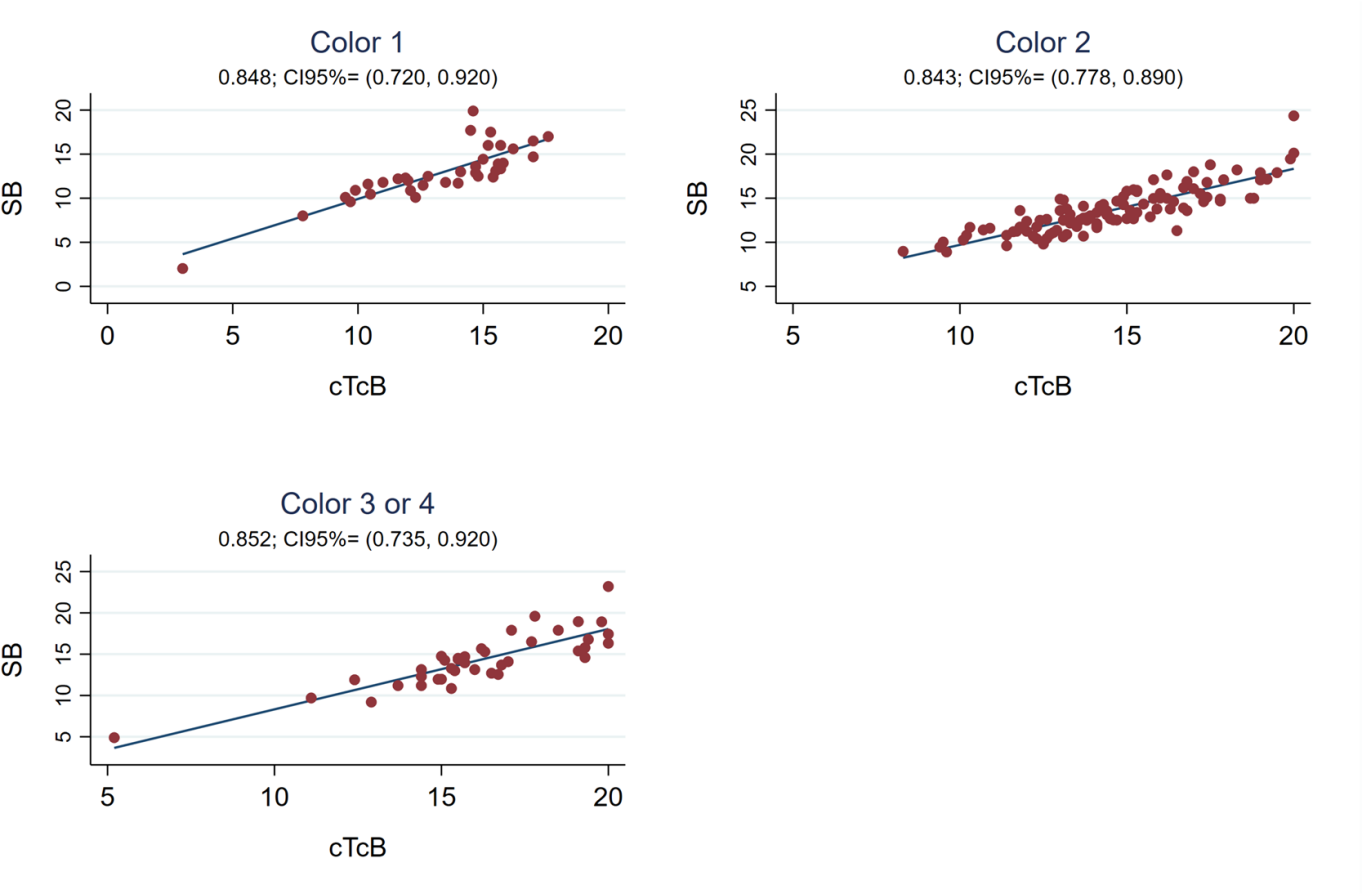
Pearson correlation coefficient between cTcB and SB depending on skin color after discontinuing phototherapy (n = 36 for color 1, n = 109 for color 2, n = 39 for color 3or4)

**Fig. 7:**
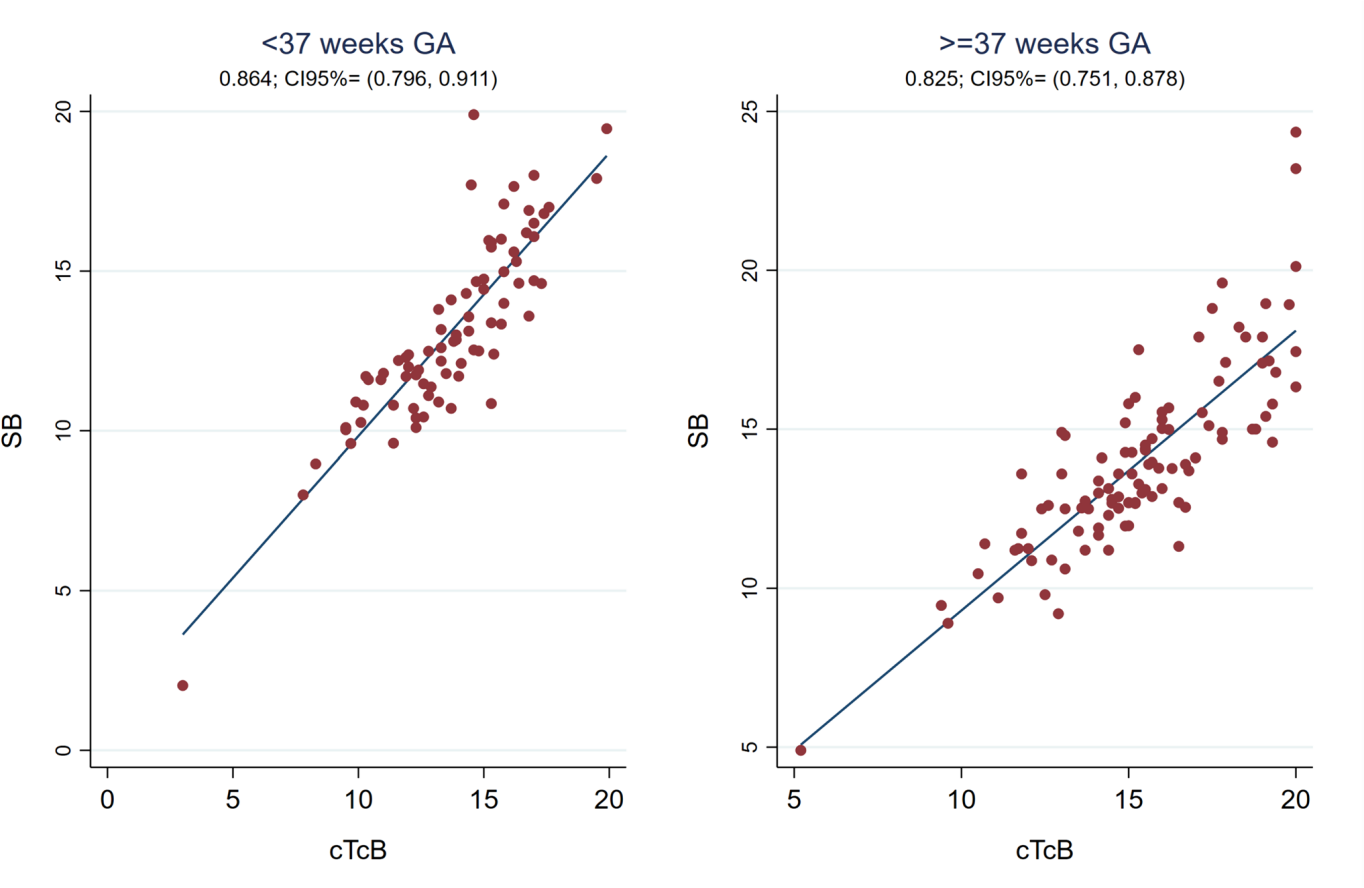
Pearson correlation coefficient between cTcB and SB depending on gestational age after discontinuing phototherapy (n = 80 for < 37 GA, n = 104 for ≥ 37 GA). *GA: gestational age*

The Pearson correlation coefficient between cTcB and SB was also strong globally after discontinuing phototherapy [r0.84 (95%CI 0.79;0.88)] (see Fig. 1), with the SB-cTcB bias Bland Altman estimation of -1.015 mg/dL (95%LOA -4.19;2.16). This correlation coefficient remained good when analyzing it depending on the hours after discontinuing treatment and on the neonate’s skin color (see Figs. 5 and 6). Bland Altman bias estimation and its LOA between cTcB and SB depending on skin color after phototherapy resulted in: -0.43 mg/dL (95%LOA -3.78;2.92) for color1, -0.91 mg/dL (95%LOA -3.74;1.92) for color2, -1.84 mg/dL (95%LOA -5.20;1.51) for color3or4. Figure 7 shows the Pearson’s correlation coefficients between cTcB and SB after phototherapy depending on gestational age: both strong for preterm (r0.86) and term infants (r0.83). Bland Altman bias estimation and its LOA between cTcB and SB depending on gestational age after phototherapy resulted in: -0.59 mg/dL (95%LOA -3.46;2.28) for preterm infants, and -1.34 mg/dL (95%LOA -4.59;1.91) for term infants.

Table 2 shows results from the multiple linear regression. The bias SB-cTcB during phototherapy was significantly associated with cTcB measurement as well as with gestational age. On the other hand, the bias SB-cTcB after phototherapy was significantly associated with cTcB measurement and skin color (only significant for color3or4). Finally, after 23 h of phototherapy, the bias SB-cTcB was significantly associated only with skin color (again, significantly associated with color3or4).

**Table 2:** β coefficients and its confidence intervals (CI) of the associations between SB-cTcB bias and cTcB, gestational age and skin color. Results during phototherapy, after phototherapy and phototherapy > 23h

	**β**	**95% CI**		**p-value**	**n**
**cTcB during phototherapy**	-0.44	-0.50	-0.38	**< 0.001**	296
Gestational age	0.19	0.11	0.28	**< 0.001**	
Color1					
1	0.00				
2	-0.02	-0.50	0.46	0.935	
3or4	-0.38	-0.97	0.21	0.207	
**cTcB after phototherapy**	-0.10	-0.19	-0.02	**0.018**	184
Gestational age	0.00	-0.11	0.12	0.943	
Color1					
1	0.00				
2	-0.36	-0.97	0.25	0.249	
3or4	-1.13	-1.92	-0.34	**0.005**	
**cTcB after phototherapy >23h**	-0.10	-0.21	0.02	0.089	131
Gestational age	0.04	-0.10	0.18	40.597	
Color1					
1	0.00				
2	-0.37	-1.11	0.37	0.322	
3or4	-1.50	-2.56	-0.44	**0.006**	

*CI* Confidence interval

## Discussion

Jaundice meters are reliable and can be used at the newborn's bedside by minimally-trained personnel with numerous reported benefits [3, 8, 9, 14, 21, 32, 33]. They measure bilirubin in subcutaneous tissue and are designed to agree as closely as possible with SB [3, 14]. Some authors have hypothesized that because bilirubin deposits in the subcutaneous fat in a similar way as in the brain, TcB could more closely estimate brain bilirubin [3]. Most clinical studies have shown an accuracy of ± 3 mg/dL between TcB and SB [3, 11, 31]. Despite being a powerful tool to clinically screen for neonatal hyperbilirubinemia, some controversies remain as to their reliability depending on exposure to phototherapy. Phototherapy eliminates bilirubin from the skin and subcutaneous tissue by producing photoisomers that are more easily eliminated. Bilirubin concentration in the skin decreases more rapidly than in blood during phototherapy, at least initially [3, 13, 17, 24, 34, 35]. Therefore, jaundice meters may be less reliable in neonates receiving phototherapy or < 24 h after interrupting it [3]. TcB measurements are not widely accepted as a surrogate measure of SB during phototherapy, although not all the authors agree in this statement [10, 13, 18, 20–23, 25, 34–37]. There seems to be a better TcB/SB correlation when TcB is measured in covered skin [13, 17, 21–24, 26–28, 35, 38–41].

In our present study, we obtained a much stronger TcB/SB correlation when measured in covered skin (r0.79) versus exposed skin (r0.54) (see Fig. 1). The cTcB/SB correlation was strong during phototherapy globally (r0.79, see Fig. 1) and for colors2 and 3or4 (r0.74–0.81), being worse for color1 (r0.67, see Fig. 3). However, correlation is lower than we reported in our previous study before initiating phototherapy (r0.91–0.94) [11]. The mean SB-cTcB bias ± 2SD obtained was -1.02 ± 3.24 mg/dL (95%CI -4.19;2.16) globally, with a wider range than reported in our previous study before phototherapy, but within acceptable limits (± 3.24 mg/dL) [11]. These results are in agreement with Tan, who found that phototherapy reduced the cTcB/SB correlation (JM-103™), but did not completely eliminate it [17]. Katayama found moderate cTcB/SB correlations (JM-103™) before and during phototherapy in a retrospective study in term Japanese neonates [37]. Rylance and Johnson also described that cTcB (JM-103™) was reliable to guide phototherapy in Black Malawian and Haitian neonates respectively [40, 42]. Castro studied cTcB/SB and eTcB/SB correlations (JM-103™) during phototherapy in a small mostly Caucasian term and moderate-preterm neonatal sample finding the cTcB/SB correlation to be relatively strong (r0.74) but with LOAs’ range too wide (-3.8;4.6 mg/dL) to use cTcB to guide phototherapy. Only Costa-Posada and Pendse used the same device as us (JM-105™). Costa-Posada found a good cTcB/SB correlation during phototherapy in Caucasian preterm and term newborns with SB-cTcB biases that rarely exceeded 2 mg/dL [28]. Pendse found a good cTcB/SB correlation in preterm Indian neonates, stating that cTcB is a good option in low-income countries with high prematurity rates and with SB measurements often unavailable [39]. Most of these studies (except Ozkan’s who used an older version of the device -JM-101™- [25]) agree with our results. But, unlike our study, these were not performed in multiethnic populations nor specified skin color. In addition to this, most of them only included term infants, except for Pendse and Huzelbos which only included preterm infants [35, 39]. Many other studies done with BiliChek drew contradictory conclusions [7, 13, 19, 22, 26, 27, 34, 41]. However, most of these studies, except Murli and Reyes´s, found cTcB useful during phototherapy. The contradictory results could be explained by different study populations, inclusion or not of preterm infants, irradiance of phototherapy, etc. [41].

The weaker correlation and wider ranges of SB-cTcB biases obtained during phototherapy could be explained by the higher levels of SB in these patients, as in most cases levels of TcB above 15 mg/dL need to be confirmed with a blood sample due to a worsening correlation [3, 11, 21, 31]. However, once a spontaneous decline in TcB is detected and maintained, the risk of subsequent hyperbilirubinemia is low [31]. The delayed decrease of cTcB relative to SB during the first hours of treatment could also contribute to the worse cTcB-SB correlation during phototherapy. Also the older age of infants undergoing phototherapy could contribute to a worse correlation, as skin pigmentation may increase with age [39, 41].

Some data show that the TcB/SB correlation worsens with decreasing gestational age, at the same time that the threshold for phototherapy decreases [5, 43]. Despite the controversies, there seems to be a strong correlation in preterm babies, that may vary depending on gestational age [7, 8, 10, 11, 13, 21, 23, 35, 39, 44–46]. In line with this last statement we found that the SB-cTcB bias during phototherapy depended on gestational age as shown in Table 2. These results agree with most authors except Zecca and Costa-Posada’s [3, 7, 13, 27, 28, 40, 42]. De Luca found a good cTcB/SB correlation (r0.84) in extremely premature infants undergoing phototherapy [7]. In our study, the cTcB/SB correlation during phototherapy was even better in preterm than in term infants (r0.80 versus r0.72) (see Fig. 4). We did not observe this dependence on gestational age after phototherapy.

Several authors have also studied the usefulness of TcB to control rebound bilirubin after phototherapy. Limited data exist about when eTcB is reliable after phototherapy, being historically recommended after 18–24 h [17]. In a prospective observational study, Grabenhenrich estimated that eTcB (JM-103™) underestimated SB by 2.4 mg/dL (SD 2.1 mg/dL) during the first 8 h post-phototherapy, giving a safety margin of 7 mg/dL below the treatment threshold to skip measuring SB. This study included mostly Caucasian newborns and none required a second phototherapy course [12]. More recently Akin observed that the best moment to measure cTcB (BiliChek™) was at least 7 h after stopping phototherapy (r0.98) [47]. Juster-Reicher obtained similar results (8 h) in Israeli neonates (r0.65–0.80) [48]. Castro found a good cTcB/SB correlation (r0.90) 12 h after phototherapy [20]. In agreement with Castro, our results show a good cTcB/SB correlation globally after phototherapy (r0.84), improving 12 h after discontinuing treatment from 0.75 to 0.91 (see Figs. 1 and 5). Our sample was much larger and multiethnic.

We reported correlations among different color groups, all of them similar (r0.84–0.85, see Fig. 6). Modern jaundice meters use specific algorithms to isolate bilirubin from other skin chromophores [3]. However, in accordance to our previous study and to other authors, the mean SB-cTcB bias varied depending on the group, being the overestimation higher and the range wider in darker-skinned newborns (see Table 2) [3, 4, 9, 11, 29, 32, 49, 50].

The most currently-used jaundice meters, BiliChek™ and JM-103/105™, have a similar performance. However, the first one needs a disposable tip for each use, increasing costs, is more time-consuming and may also underestimate SB at higher levels (> 15 mg/dL) [3, 6, 31]. Clinically speaking, it is safer to slightly overestimate SB than underestimate in order to avoid misclassification of newborns requiring phototherapy.

To the best of our knowledge, ours is the first reported prospective study analyzing both cTcB and eTcB reliability during and after phototherapy in multiethnic populations of term and preterm newborns according to skin color. Classifying newborns by ethnic group is complex, imprecise and unreliable, due to the variability of skin tones within an ethnic group. We used a reliable validated neonatal skin color classification [11, 29].

Our study has limitations. First, we could not recruit many patients belonging to color4 given the characteristics of our population. Second, we only used one device (JM-105™^)^, for which our results may not be generalizable to other jaundice meters. Last, we collected data at a single center, therefore our results may not be generalizable.

## Conclusion

The cTcB/SB correlation was strong for term and preterm infants after phototherapy treatment and, to a lesser extent, during treatment. The SB-cTcB bias estimation fell within acceptable limits during phototerapy (± 3.24 mg/dL). However, eTcB only correlated well with SB after 24 h of finalizing phototherapy.

Our study supports the reliability of cTcB to assess SB during and after phototherapy in multiethnic populations of preterm and term babies, with statistical differences among skin color after treatment and on gestational age during treatment. This may help reduce the number of blood samples, thus reducing both newborn pain and economic costs during jaundice treatments. A larger sample for color4 may increase reliability of cTcB in this group.

### Supplementary Information

Below is the link to the electronic supplementary material.

Supplementary file1 (DOCX 200 KB)

Supplementary file1 (DOCX 106 KB)

Supplementary file1 (DOCX 105 KB)

## Data Availability

The datasets generated and/or analyzed during the current study are available from the corresponding author on reasonable request.

## Abbreviations

SB: Serum bilirubin
TcB: Transcutaneous bilirubin
cTcB: Transcutaneous bilirubin in covered skin
eTcB: Transcutaneous bilirubin in uncovered or exposed skin
SD: Standard deviation
LOA: Limits of agreement
