# Supplementary material for: Transcutaneous bilirubin reliability during and after phototherapy depending on skin color
Source: Eur J Pediatr. 2024 Apr 6;183(7):2819–30. doi: 10.1007/s00431-024-05516-4 (PMC11192662; doi:10.1007/s00431-024-05516-4)
Supplement: Supplementary file 1 — Supplementary file1 (DOCX 200 KB) [file 431_2024_5516_MOESM1_ESM.pdf]

Appendix 1. Neomar's neonatal skin color scale.[34]

Color1 (light)	Color2 (medium-clear)	Color3 (medium-dark)	Color4 (dark)

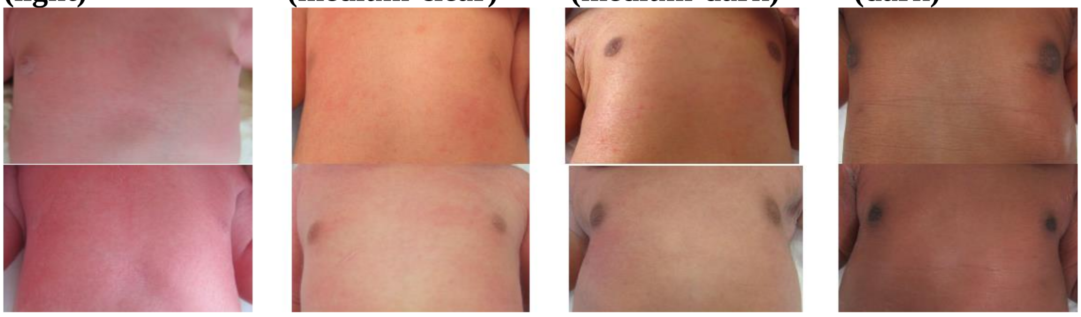

White/pink skin	Pink/beige skin	Light brown skin	Brown skin
