# Supplementary material for: Transcutaneous bilirubin reliability during and after phototherapy depending on skin color
Source: Eur J Pediatr. 2024 Apr 6;183(7):2819–30. doi: 10.1007/s00431-024-05516-4 (PMC11192662; doi:10.1007/s00431-024-05516-4)
Supplement: Supplementary file 2 — Supplementary file1 (DOCX 106 KB) [file 431_2024_5516_MOESM2_ESM.pdf]

Appendix 2. Data collection sheet.

Newborn's medical record number / Newborn's name and surnames:	Mother's medical record number / Mother's name and surnames:
Gestational age (weeks [days]):	Mother's birth country:
Weight (g):	Breastfed / formula-fed
Weight at discharge (g):	Newborn's blood type:

1. Informed consent: yes / no

2. Assignment of skin color group at 24 hours of life:

Color (visual scale)
1 2 3 4

3. Placing of a patch in the mid-sternal area

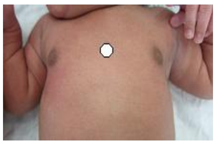

2. Phototherapy: **Beginning:** **hours of life**
End: **hours of life**
Rebound SB: **hours of life**

3. Measurement of TcB in exposed skin (eTcB) / in unexposed skin (cTcB) with each SB measurement [maximum 20 minutes between SB/TcB]

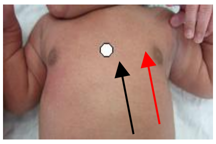

Hours of life	cTcB	eTcB	SB
…… hours			
…… hours			
…… hours			
…… hours			
…… hours			
