# Supplementary material for: Transcutaneous bilirubin reliability during and after phototherapy depending on skin color
Source: Eur J Pediatr. 2024 Apr 6;183(7):2819–30. doi: 10.1007/s00431-024-05516-4 (PMC11192662; doi:10.1007/s00431-024-05516-4)
Supplement: Supplementary file 3 — Supplementary file1 (DOCX 105 KB) [file 431_2024_5516_MOESM3_ESM.pdf]

**Informed consent**

**Study title: RELIABILITY OF TRANSCUTANEOUS BILIRUBIN DETERMINATION ACCORDING TO SKIN COLOR AND PHOTOTHERAPY EXPOSURE IN COVERED/UNCOVERED SKIN**

**Main investigators: Júlia Candel-Pau (Assistant physician of the Neonatal Unit of Hospital del Mar, Barcelona) and María Ángeles López-Vílchez (Head of Pediatrics, Hospital del Mar, Barcelona).**

I (first name and surnames) ________________________________________________

As (relationship to the participant) ____________________________________________
Of (name of the participant) ________________________________________

I have read the consent form that has been given to me.

I could ask questions about the study. I have received satisfactory answers to my questions.

I have received enough information about the study.

I have spoken with _________________________________ (investigator's name)

This study will not increase the number of venous punctures nor will entail any risk for my son / daughter, and it only involves a minimal increase of the blood drawn (an extra 0.3 mL).

The Pediatric Department of Hospital del Mar guarantees confidentiality of all personal and medical data about me and my son / daughter.

I understand that my participation is voluntary.

I understand that I can withdraw from the study:

1. Whenever I want.
2. Without having to give explanations.
3. With no impact on my son's / daughter's medical care.

I freely give my consent for _______________________________ (name of participant) to participate in the study.

I have received a signed copy of this informed consent.

________________ ________________________________

Date Participant signature (legal guardian / parent)

I have explained the nature and purpose of the study to the above persons.

________________ ________________________________

Date Investigator's signature

Barcelona, __________________ (date)
